# Biosafety and Biosecurity Challenges Facing Veterinary Diagnostic Laboratories in Lower-Middle Income Countries in Southeast Asia: A Case Study of Thailand

**DOI:** 10.1177/1535676019869771

**Published:** 2019-08-19

**Authors:** Jarunee Siengsanan-Lamont, Somjai Kamolsiripichaiporn, Soiratchaneekorn Ruanchaimun, Tuangthong Patchimasiri, Banjong Jongrakwattana, Stuart D. Blacksell

**Affiliations:** 1 Animal Health and Laboratory Consultant, Perth, WA, Australia; 2 Animal Health and Laboratory Consultant, Bangkok, Thailand; 3 Mahidol-Oxford Tropical Medicine Research Unit, Faculty of Tropical Medicine, Mahidol University, Bangkok, Thailand; 4 National Institute of Animal Health, Bangkok, Thailand; 5 Nuffield Department of Clinical Medicine, Centre for Tropical Medicine & Global Health, University of Oxford, Oxford, UK

**Keywords:** biosafety, biosecurity, challenges, veterinary laboratory, Thailand

## Abstract

**Introduction:**

Global concerns over emerging and transboundary infectious zoonotic diseases have increased disease diagnostic demands, especially in the veterinary sector. In developing or newly developed countries where the sector often works under limited capacity, biosafety and biosecurity are unlikely to be high-priority issues. A recent development program supported by the Biological Threat Reduction Program of the Defense Threat Reduction Agency funded by the US government aimed to increase biosafety and biosecurity measures of government veterinary diagnostic and research laboratories in Thailand.

**Objective:**

The purpose of this article is to identify biosafety and biosecurity challenges, opportunities, and recommendations.

**Methods:**

Eleven government laboratory centers were assessed against the *Biosafety in Microbiological and Biomedical Laboratories* (*BMBL*) biosafety level 2 (BSL-2) requirements checklist. The *BMBL* assessment outcomes were then combined with the outcomes of discussion sessions, and the results of pre- and post-test questionnaires conducted during biosafety assessment workshops and self-evaluation reports using the Food and Agriculture Organization Biosafety Laboratory Mapping Tool of each laboratory center were reviewed and summarized.

**Results:**

Despite established national policies on laboratory biosafety and biosecurity, major challenges included (1) harmonization and enforcement of these policies, especially at the regional level, and (2) engagement of personnel in implementations of biosafety and biosecurity measures.

**Conclusion:**

Consistent biosafety policy and allocated resources together with regular training are required to develop sustainable biosafety and biosecurity at the national level. Collaboration between regional countries, international organizations, and donors is essential for improving biosafety and biosecurity on a global scale through setting regional priorities, enacting regulatory standards, and providing technical and financial support.

Increasing global concern over emerging infectious diseases, especially zoonotic diseases (eg, Ebola, Nipah, avian influenza, etc) has directly affected the veterinary sectors. More than 75% of the emerging infectious diseases and 60% of communicable diseases that infect humans are zoonoses.^1^ Veterinary diagnostic laboratories are often at the forefront of detecting these potentially pathogenic diseases.^2^ This aspect has raised concerns over the biosafety and biosecurity capacities of veterinary diagnostic laboratories, especially those in developing countries where resources are commonly limited. It has been recognized that biosafety and biosecurity implementation are likely compromised in underresourced laboratories.^3,4^

This article reviews lessons learned during recent activities supported by the Biological Threat Reduction Program of the Defense Threat Reduction Agency (BTRP-DTRA) funded by the US government using a case study of Thailand under the Thailand Veterinary Laboratory Capacity Building Project (known locally as “CATH2”) operating between 2017 and 2019. The report provides a summary of the findings on biosafety and biosecurity challenges and opportunities facing veterinary diagnostic and research laboratories, and it provides recommendations for future programs in other resource-constrained settings. The laboratory operation and mentoring activities were conducted by the Mahidol-Oxford Research Unit (MORU) between 2015 and 2019 in Lao PDR, Cambodia, and Thailand. The primary objective of the programs was to enhance biosafety, biosecurity, and biosurveillance capabilities at the national and regional levels.

## Methods

### Scope of the Study

**Figure 1.**
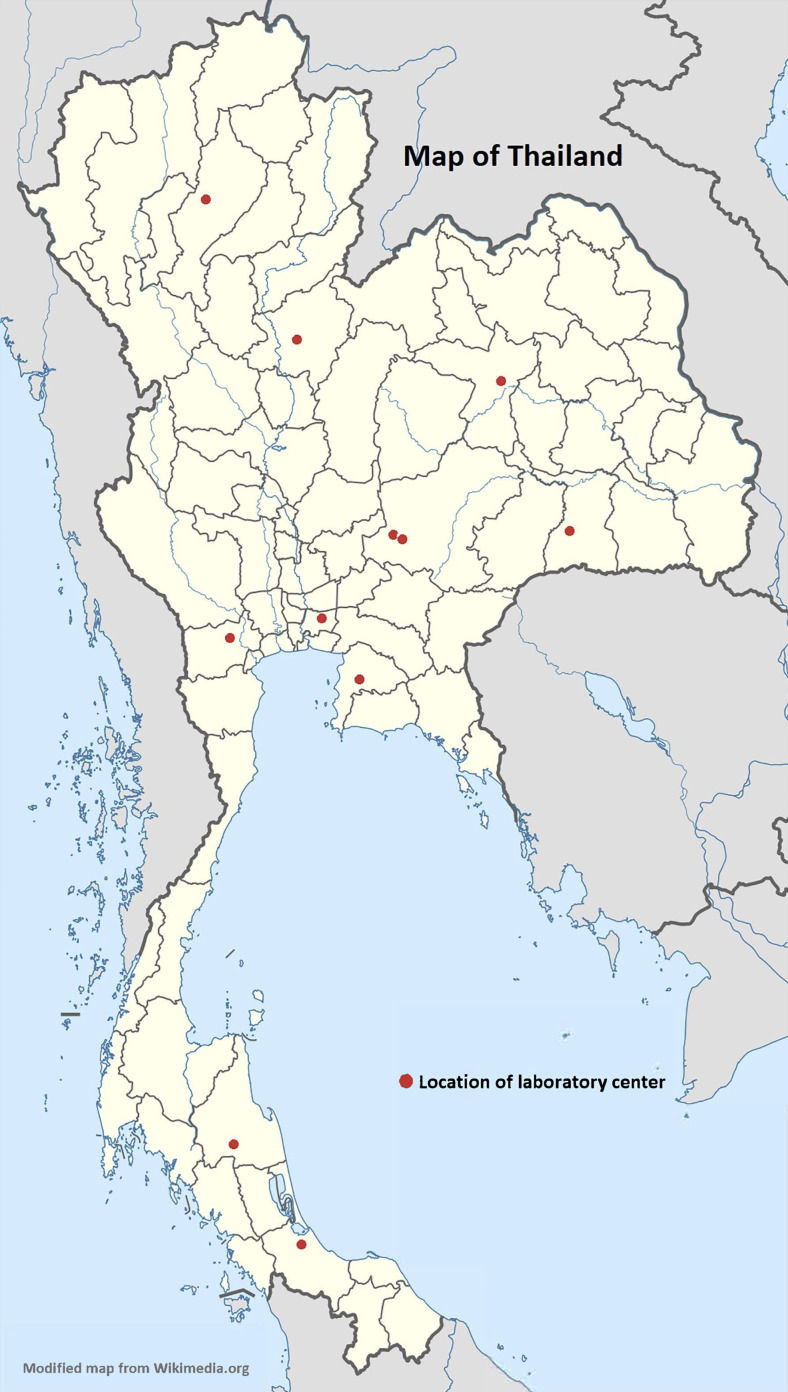
Locations of the 11 laboratory centers included in this study.

All 11 government veterinary diagnostic and research laboratory centers in Thailand under the Department of Livestock Development, Royal Thai government, including central, regional, and reference laboratory centers were enrolled in this study. Locations of these centers are shown in Figure 1. The activities of the laboratory biosafety training and mentoring component of the CATH2 project included a 2-week training of trainers (TOT). The TOT course organized in August 2017 at the central laboratory institution focusing on the biosafety and quality assurance officers (mostly veterinarians and/or scientists) from all laboratory centers was delivered by the MORU biosafety team and an outsource educator team. The course aimed not only to provide training on the principles and practical knowledge on applications of biosafety, biosecurity, and biosafety assessment, but also to equip these officers with teaching skills for knowledge transfer to their peers.

The biosafety assessment workshop was then requested by the laboratory biosafety committees to be conducted on site at every laboratory center for all staff to attend. The 1-day biosafety assessment workshops were delivered by the MORU biosafety team at each laboratory center, with the primary objectives of introducing risk assessment and mitigation concepts to improve biosafety and biosecurity using facilitated discussion sessions to encourage critical thinking among participants.

### Data Collection and Analyses

There were 3 main components of data collection and analyses in this study: (1) qualitative data collected during the laboratory biosafety assessment and discussion, (2) a quantitative analysis of the biosafety assessment workshop pre-test and post-test outcomes, and (3) an analysis of the self-evaluation results using the Food and Agriculture Organization (FAO) Laboratory Mapping Tool for Safety (LMT-S). Key findings of each component including challenges, opportunities, and recommendations are identified, summarized, and discussed in this article.

#### Laboratory biosafety assessments

**Figure 2.**
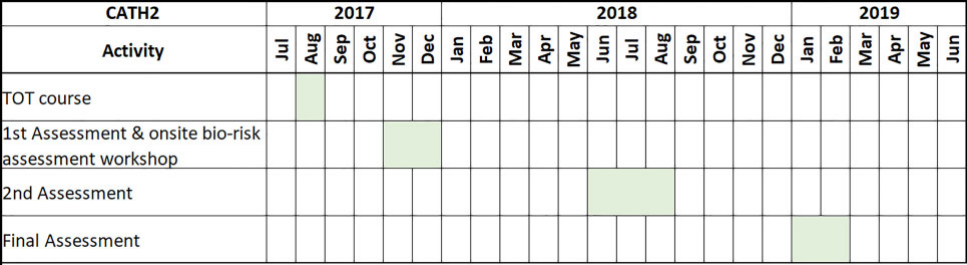
CATH2 mentoring and assessment activity Gantt chart.

The assessments were conducted on 3 occasions at most laboratories of these 11 centers using the Centers for Disease Control and Prevention, *Biosafety in Microbiological and Biomedical Laboratories*, 5th edition (*BMBL*), checklist against the Biosafety Level 2 (BSL-2) criteria.^5^ The first assessments were completed during November to December 2017, the second assessments from June to August 2018, and the final assessments from January to February 2019. After each assessment, laboratories were provided with a report of the corrective action requests (CAR), gaps, and recommendations for improving biosafety and biosecurity measures. Each laboratory center was given the opportunity during discussion sessions to explain their standard biosafety practices, situations, and constraints before agreeing to the report. The team then followed up CAR progress and organized BSL-2 certification for eligible laboratories when fully compliant. Gaps, recommendations, and discussion outcomes were qualitatively analyzed. A Gantt chart of the CATH2 mentoring and assessment activities is presented in Figure 2.

#### Assessment of the biosafety workshops

During the first assessment, an on-site biosafety assessment workshop was conducted at each laboratory as requested by the laboratory biosafety committees. Participants were encouraged to participate in discussion sessions during the workshop on risks associated with their daily work and outcomes reviewed as a part of qualitative data together with laboratory biosafety assessment data. Pre- and post-training tests with the same set of questions on biosafety, biosecurity, risk assessment, management concepts, and principles were used to evaluate the participant’s background and the efficacy of the biosafety assessment workshop. The questions included in the test are presented in Table 1. The answer sheets were marked and scored by a facilitator. Participants were also asked to provide feedback for future improvement of the workshop. A summary of the results was reported back to the participating laboratories for their records and used for the quantitative analysis in this study. The pre- and post-test results were analyzed using the Shapiro-Wilk test for normality,^6^ the Wilcoxon signed-rank test,^7^ and the Kruskal-Wallis test.^8,9^

**Table 1.** Example of Pre- and Post-test Assessment Questions.

Pre- and Post-test Questions
Describe in your own words the definition of:
Biorisk management
Biosafety
Biosecurity
Biosafety and biosecurity risk assessment
Risk mitigation
Explain the importance and implementation of biosafety and biosecurity risk assessment and mitigation

#### Self-evaluation using the FAO LMT-S tool

The FAO LMT-S (version 2016) can be downloaded from the FAO webpage (http://www.fao.org/ag/againfo/programmes/en/empres/news_231216b.html). In February 2016, a Thai national LMT training workshop was held followed by a regional training held in August 2016 with support from FAO, DTRA, and MORU to assist with regional implementation.^10^ The tool is categorized into 4 areas: administration, operational, engineering, and personal protective equipment (PPE).^10^ The evaluation areas with their categories and subcategories included in the tool are listed in Table 2. During the self-assessment, the laboratory biosafety team chooses a score between 1 (lowest/ minimum) and 4 (highest/ fully implement) for each subcategory that best describes their current situation. Details of the tool and the evaluation process are described by Mouillé et al^11^ and Blacksell.^12^ Results of the FAO LMT-S self-evaluation of 2016, 2017, and 2018 of each laboratory center were analyzed using the Wilcoxon signed-rank test^7^ to identify changes of mean scores over time. The R Studio statistical package (version 3.6.0/64 bit)^13^ was used for these statistical analyses.

**Table 2.** The Laboratory Mapping Tool for Safety Evaluation.

Area	Category	Subcategory
Administration	General	General biosafety
		General security
		Biosafety audits
		Risk assessment
		Pest control
	Personnel health and safety	Medical (occupational health) surveillance program
		Vaccination/prophylaxis
		Emergency documents and emergency response supplies
		Formal program for accidents, adverse incidents
	Training and competency	Biosafety training availability
		Biosafety training objectives
		Staff management and training (specific to agents)
		Training and competency for emergency response
	Biosafety manual/standard operating procedures (SOPs)	Biosafety manual
		Biosafety requirements included in SOPs
Operations	Good laboratory practices	Evidence of Good Laboratory Practices (BSL-2 minimum practices)
		Good Laboratory Practices enforcement (BSL-2 minimum practices)
		Laboratory housekeeping
		SOPs for cleaning and disinfection
		Disinfectant use and labeling
		Biological or chemical indicators (autoclaves)
		Biosafety equipment maintenance
	Containment	Risk assessment for biocontainment
		Access security measures
		Training and competency for level BSL-2 agent manipulation
		Biohazard signage (containment BSL-2 level)
		Potentially infectious samples manipulation (level BSL-2)
		Emergency response plan in case of major failure (BSL-2 level)
	Containment biosafety level 3	Infectious samples handling in a BSC (BSL-3 level)
		Biohazard signage (containment BSL-3 level)
		Training and competency for level BSL-3 agents manipulation
		Facility manager BSL-3 operations
		Certification (international or national regulations) for BSL-3 operations
		Annual maintenance plan for the BSL-3 laboratory
		Directional negative-pressure air flow for BSL-3 ventilation
		Supply and exhaust air filter for BSL-3 ventilation
	Waste disposal	Waste disposal containment and rendered noninfectious
		Incinerator
		Waste management
		Sharps for disposal
		Equipment and disposable materials availability
	Shipping of infectious substances	Specimen reception and distribution
		Training and competency for infectious agent shipment
		Packaging of infectious materials
		Records of infectious agent shipment
		Reusable secondary container for shipment
	Animal facilities	Experiment or animal facility accreditation
		Staff accreditation for animal care and use
		Animal ethics committee
		Animal waste decontamination
		Equipment for animal waste disposal
		Medical (occupational health) surveillance program for staff working with animals
		Specialized PPE for experimental animal facility
Engineering	Premises	Premises’ biological quarantine requirements
		Local and national regulations for premises knowledge and enforcement
		Premises’ comfort and level of quality
		Work areas including benching and illumination quality
		Handwashing sink
		Access to lockers or storage shelves
		Necropsy
	Chemical hazard containment	Separation of chemicals
		Compressed gases
		Liquefied gases
		Radiation: personnel protection and physical protection
		Radiation protection officer
		Radiation spill kit
	Chemical security	Chemical waste
		Chemicals storage
		Chemical safety officer
		Chemical spill kit
	Emergencies	Emergency response/exercises (fire drills, spill cleanup)
		Emergency procedures (shower)
		Biological spill kits availability
		Emergency eyewash
	Fire hazard	Fire detection and suppression system
		Fire alarms and fire drills
		Fire evacuation plan, fire exits
		Fire extinguishers
	Electrical	Electrical equipment approval
		Electrical equipment testing
		Electrical earthing or grounding
		Response plan for power failures
	Biological safety cabinet	BSC testing
		BSC use
		BSC conformity
PPE	General situation	Risk assessment for PPE requirement
		Risk assessment for glove selection
		Availability of PPE
		PPE training
	Use of PPE	PPE usage and removal
		PPE (protective eyewear or face protection)
		Common object handling in the work area
		Use of PPE when working with temperature extremes
	PPE disposal	Reusable PPE maintenance program
		Reusable PPE cleaning procedures
		Laundry practices
		Disposable gloves usage
		Decontamination (disposal) of nonreusable PPE

Abbreviations: BSC, biosafety cabinet; BSL, biosafety level; PPE, personal protective equipment.

## Results

### Laboratory Biosafety Assessments and Discussion Outcomes

#### General findings

Most of these laboratories were responsible mainly for disease diagnoses and research of major animal diseases and animal product quality assurances. Diagnostic activities involved various pathogens including parasites, fungi, bacteria, viruses ranking from the risk group 1 (eg, *Escherichia coli* ^14^) through to the risk group 3 (eg, *Brucella abortus*, *Burkholderia pseudomallei*, Highly Pathogenic Avian Influenza [HPAI]m H5N1, etc^14^). Selected laboratory centers have specific tasks responsible for veterinary vaccine quality assurances, and in one case, serving as a World Organization for Animal Health (OIE) Southeast Asian Regional Reference Laboratory Center for Foot and Mouth Disease (FMD RRL). All of the laboratory centers have ISO17025 (testing and calibration laboratories)^15^ accreditations for a number of diagnostic tests. Only 5 of these centers received accreditation for ISO9001:2015 (quality management systems).^15^ All laboratories were categorized as biosafety level 1 and 2 facilities. Biosafety level 3 (BSL-3) biocontainment facilities were installed in most regional laboratory centers during the era of HPAI H5N1 outbreaks when funding was abundant. However, those BSL-3 laboratories were no longer in operation because of unaffordable maintenance and running costs, except for the BSL-3 facility at the FMD RRL because of the necessity of providing FMD diagnostic services to member countries in Southeast Asia.

#### BSL-2 certification

Outcomes of the assessments revealed that of 33 laboratory rooms that applied for the BSL-2 certification, 16 met the *BMBL* BSL-2 criteria checklist (48% of 33 requested laboratory rooms) and were awarded BSL-2 certification in March 2019. While some rooms required only minor corrective actions to be eligible for the BSL-2 certification, many facilities needed major changes to their infrastructure, workflow, and practices.

### Gaps and Challenges Identified During the Assessments

The biosafety and biosecurity laboratory assessment activity revealed that all laboratory centers generally had sufficient and practical equipment and facilities. Most government buildings had a similar blueprint that had not been specifically designed to accommodate laboratory facilities. While some buildings may have been designed for laboratory purposes, it proved difficult to upgrade and/or modernize. Like government sectors elsewhere, significantly large budgets for refurbishment or alteration of the infrastructure were hard to get approved, and the process was often slow and complicated. Essential safety and emergency equipment including biosafety cabinets (BSCs), chemical fume hoods, laminar flow cabinets, fire extinguishers, PPE, were provided in all centers but varied in numbers depending on each center’s policy and budget prioritization. Only some centers had extra safety features (eg, emergency showers and eyewash, smoke detectors, fire alarms, and limited entry systems [key card access]). Operational budget constraints also resulted in insufficient maintenance funding (for some existing equipment such as BSCs) and inadequate safety feature budgets (eg, N95 masks, earthing systems, key card system, fire/smoke alarms, etc).

Despite established policies on laboratory biosafety and biosecurity including the Thailand Pathogens and Animal Toxins Act, B.E.2558 (2015),^16^ harmonization and enforcement of these policies nationwide remain major challenges. A national biosafety framework that required the establishment of a biosafety committee and an assigned biosafety officer at each laboratory center was implemented by these laboratory centers. Individual biosafety management and staff commitments varied depending on the center’s priority and policy. When mentioning biosafety and biosecurity issues, most centers focused on documentation of relevant standard of operations (SOPs) and purchasing and maintaining safety equipment specific to the ISO accreditation criteria, whereas good laboratory practices, safe workflows, security systems for storing and accessing hazardous pathogens, and biohazard waste management often received less attention. SOPs of these lower priority tasks varied between laboratories depending on resource availability. Biosafety and biosecurity trainings (eg, PPE selection, BSC operation and maintenance, risk assessments, etc) were often organized and conducted at the central laboratory institution for mostly technical/management level staff (ie, veterinarians and scientists). One-off training was often attended by different staff from regional laboratory centers, which neither covered a broad aspect of biosafety and biosecurity nor offered a continuous learning environment. In-house training at the laboratory centers on biosafety and biosecurity applications was sporadic and not available for all level staff. Staff were often vaccinated against pathogens specific to their assigned work (eg, seasonal flu vaccination for virology staff, rabies vaccination for pathology and virology staff), however coverage was not 100%.

Another major challenge identified during the training and mentoring activities was engagement of staff in the implementation of biosafety and biosecurity measures. Even though medium- to high-level technical staff demonstrated good understanding of biosafety and biosecurity applications, low-level operational staff, especially those who had limited educational background, often received on-the-job training covering their assigned technical tasks (eg, sample collection and distribution, waste transfer, etc) and/or supporting roles (eg, autoclave and cleaning infectious equipment, etc). This type of training was unlikely to encourage enhanced understanding of biosafety and its application and the risk of pathogen exposure associated with their daily works. As a result, unsafe practices and inappropriate behaviors were observed in most facilities. Examples of these practices included storing of personal belongings including food and drink inside laboratories, use of mobile phones when processing samples, incorrect choice of PPE (eg, wearing a hygienic mask when conducting HPAI diagnostic work), poorly designated clean-dirty zones, no decontamination protocol for waste bags, incorrect BSC operation, and so forth. There was also no report on potential pathogen exposure or laboratory or occupationally acquired infection at any of these laboratories. Only a few accident reports were filed in a couple of the 11 laboratory centers. There was also no SOP on postexposure prophylaxis protocol. Enforcement of the biosafety and biosecurity policies and consistent support from the senior management level are required to advocate the importance of safe practices.

### Risk Assessment Workshop Outcomes

**Figure 3.**
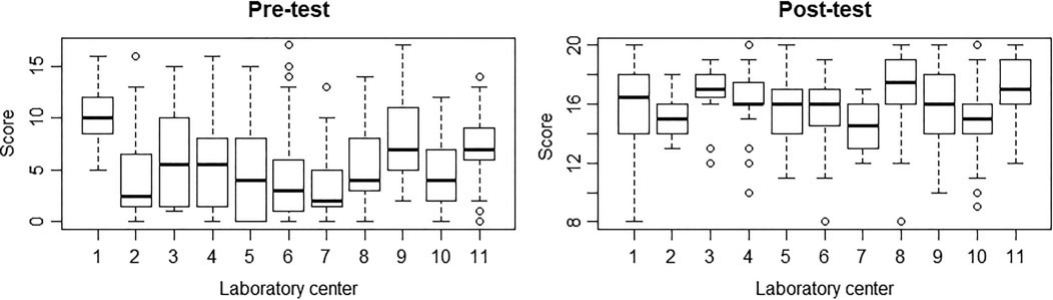
Comparison of pre- and post-test scores.

The total number of staff members who attended the risk assessment workshops was 492 and ranged across technical backgrounds, with 437 staff members completing the pretest and 399 completing the post-test. Most staff attending the workshop were categorized as scientists (35.6%) and veterinarians (11.2%). A summary of participants’ job titles is shown in Table 3. Of a total of 380 workshop participants, 97.6% submitted a feedback form indicating they agreed that the workshop was useful and/or would be able to apply the techniques to their daily work. The Wilcoxon signed-rank test of the mean scores between pre- and post-tests revealed that participants had a significantly increased understanding of biosafety and biosecurity principles and application of risk assessments to improve work safety (*P* < .001; 95% confidence interval [–10.499, –9.500]). Analysis of variance using the Kruskal-Wallis test demonstrated that the mean pre- and post-test scores between these centers differed significantly (*P* < .001 for both pre- and post-scores; Figure 3).

**Table 3.** Workshop Participant Job Titles.

Position	No.	%
Scientist	175	35.57
Veterinarian	55	11.18
Laboratory technician	54	10.98
General office staff	27	5.49
Visiting trainee	27	5.49
Housekeeper	23	4.67
Animal husbandry officer	18	3.66
Maintenance personnel	8	1.63
Gardener	5	1.02
Driver	4	0.81
Electrician	4	0.81
Director	3	0.61
Environmental scientist	3	0.61
Security	3	0.61
Para-veterinarian	2	0.41
Unclassified	81	16.46
Total	492	100.00

### Results of the LMT-S

**Figure 4.**
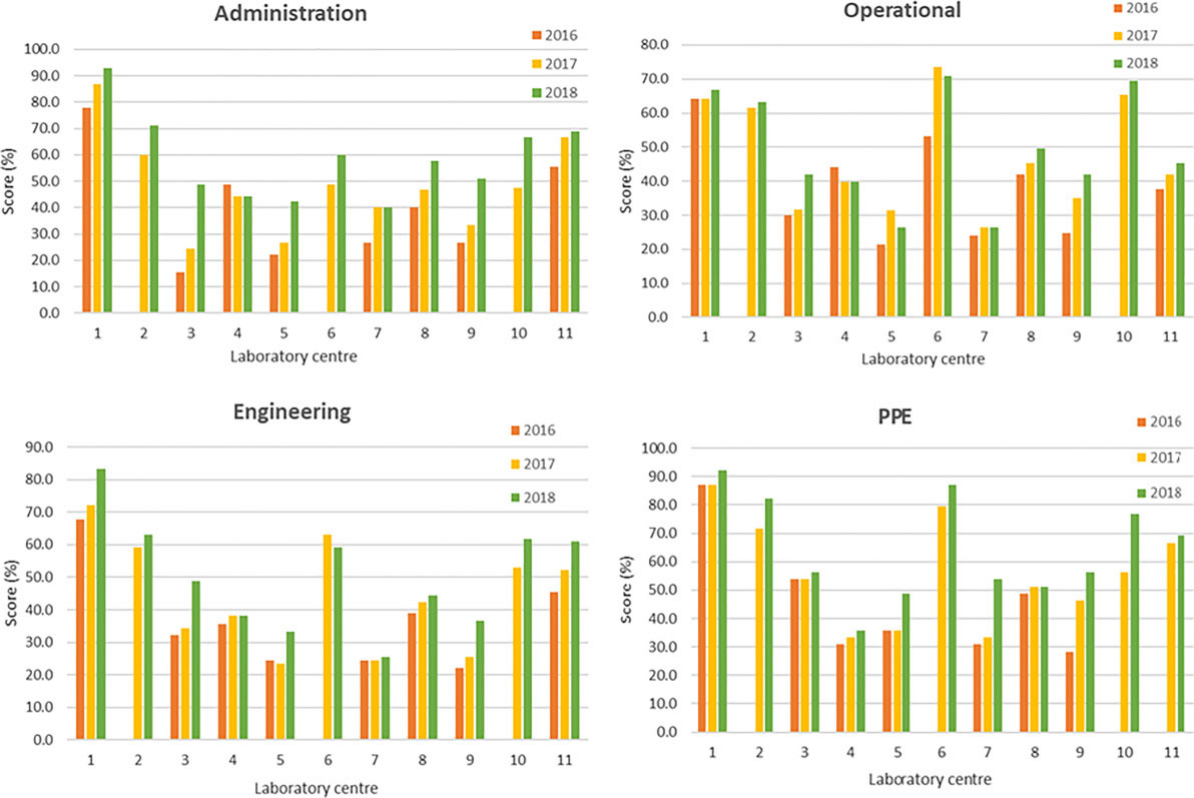
Summary of Laboratory Mapping Tool for Safety results of 2016, 2017, and 2018 for all 11 Thai veterinary laboratories.

**Figure 5.**
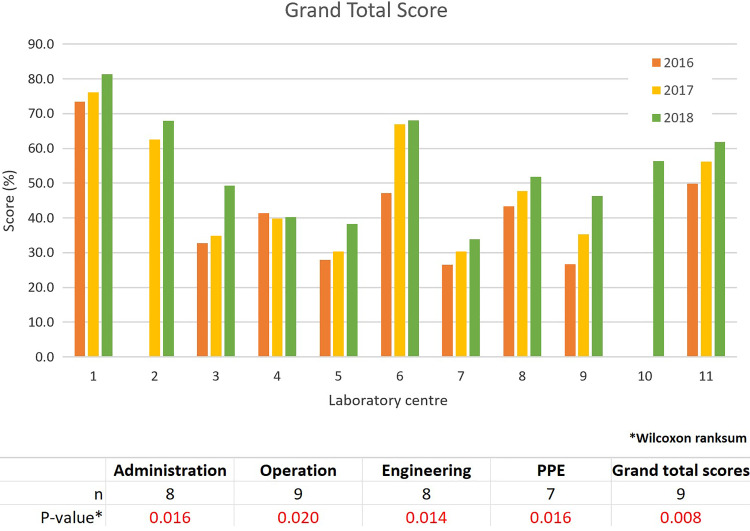
Overall LMT-S scores for the 11 Thai veterinary laboratories.

The bar charts comparing the annual area scores of all laboratory centers are presented in Figure 4. Based on the graphs, the scores in all 4 areas increased from 2016 to 2018 during the CATH2 operational period. The plots of the grand total scores (combining all 4 areas) and the Wilcoxon signed-rank test results are presented in Figure 5. The 2018 scores of all 4 areas and the grand total were significantly higher than those in 2016 (*P* < .05).

## Discussion

Lessons learned from the CATH2 program, including challenges, opportunities, and recommendations, are summarized in Table 4. In the case of Thailand, the government-operated veterinary diagnostic and research laboratories had established policies, sufficient biosafety and biosecurity equipment, and facilities with practical numbers of personnel. Even though most infrastructure had proven difficult to upgrade, it was possible to improve biosafety and biosecurity by adjusting the layout and workflows and implementing appropriate SOPs. For example, designated clean-dirty (office-laboratory) zones could be applied in the cases of limited space. It is critical that senior management staff and policy makers understand the importance of biosafety and biosecurity, not only for standard accreditations but also for the well-being of operational staff and the environment. Developing a sustainable biosafety capacity requires strong continuous policies and financial supports at the national and international level. Development of a regional framework on biosafety and biosecurity would provide guidelines and standards at the national level and advocate for financial support from policy makers.

**Table 4.** Biosafety and Biosecurity Challenges, Opportunities, and Recommendations.

Major Biosafety and Biosecurity Challenges	Recommendations for Future Program
Harmonization and enforcement of established biosafety and biosecurity policies nationwide and consistent support from the senior management levels Budget constraints resulting in insufficient maintenance of safety equipment and compromised safety features Insufficient staff awareness and engagement on biosafety and biosecurity measures, especially at the operational levels; good laboratory practices were not strictly enforced as these were not considered as one of the top priorities	Strongly and continuously advocate the importance and necessity of biosafety and biosecurity measures at the national and international levels to ensure continuity of policies and financial supports Establish of a regional/international collaboration on biosafety and biosecurity to provide technical and financial supports for countries in need and to set up a regional guidelines or standard that could be adapted and implemented at the national level Continuously support developments of sustainable capacity through standardization activities such as BSL-2 certification, ISO accreditation, proficiency testing, laboratory twinning program, etc Encourage changes of the organizational culture through regular in-house trainings and workshops using critical thinking activities on multifactorial risk-based approaches (recommended topics include biosafety and biosecurity principles and practices, selection and uses of personal protective equipment, risk assessment and mitigation, etc) Harmonize and encourage better understanding of the biosafety and biosecurity measures across the board via a national standard and/or regulatory requirements Provide financial and technical support for setting up and installing safety features and equipment where appropriate
Opportunities to further improve biosafety and biosecurity capacity	
Establish safety committee with assigned biosafety officer Adequate and functional biosafety and biosecurity infrastructure, equipment, and workforce Existing standardization activities (eg, ISO accreditations) that provide foundation skills of documentation, strategic planning and standard of operations Staff members enthusiastically and actively participate in critical thinking activities to improve their safety Increasing support of senior management officers Continuing policy and financial supports to improve biosafety and biosecurity measures are critical for a long-term sustainability Biosafety plan and manual should be revised annually; drills and exercises should be practiced regularly to raise staff awareness in biosafety implementation and to identify weaknesses for improvement The biosafety manual should be specific to each laboratory/department and tasks	Risk assessment should be practiced regularly by all staff members to engage them in critical thinking leading to behavior changes. Occupational health and safety program should be identified and standardized; pre-post exposure protocol and medical surveillance plan should be drafted and implemented Policy and financial supports to improve/upgrade infrastructure related safety features: Electrical: Even though all centers have an emergency electrical system (generators and automatic transfer switch), an uninterruptible power supply may not be available for all; an unstable electrical supply could still affect and shorten the life of expensive equipment; installation of an uninterruptible power supply and electrical stabilizer system including a budget for maintaining the system need to be considered Access control system (key card) Smoke alarm/detector Landline telephone system Grounding system: some laboratory buildings do not have a grounding system Lack of pest control such as birds, termites, ants, etc

Laboratory skills and knowledge of scientists, veterinarians, para-veterinarians, and technicians were undoubtedly competent, as proven by their ability to handle a variety of samples and diagnostic tests at a high volume and deliver accurate results. However, working with animal samples that may contain emerging infectious or zoonotic pathogens requires an understanding of associated risks and full awareness to prevent potential contaminations. Operational staff, especially those with a non-technical background, would appear to have higher risks of exposure because of the lack of introductory training on biosafety and biosecurity application and biosafety management. As inappropriate behavior of staff was observed at most facilities, risks of pathogen contamination to both staff and the environment could be considered medium to high level. The outcome of such practices could be a catastrophe if specimens contain a contagious emerging pathogen. A safety induction training covering biosafety and biosecurity principles with an assessment of competencies^17^ needs to be implemented for all new, and perhaps existing, staff. Similar to other developing countries in the region, there is no national regulatory requirement to report potential exposure of pathogens or incidence of Laboratory Acquired Infection (LAI) cases.^18^ As a result, there was no mechanism for the reporting of laboratory or occupationally acquired infections in these laboratory centers. This may be due to a combination of stigma around admitting to wrongdoing^3^ and unrecognized infection caused by low pathogenic pathogens with mild symptoms. This underreporting culture had helped encourage the false belief among staff that laboratory work has little to no risk. A system to report accidents and incidents regulated at the national level could be used in evidence-based policy making.^19^ Implementation of serosurveillance and health-monitoring programs of laboratory personnel could also help to raise awareness among staff.

Organizational culture, together with lack of staff engagement, could also pose risks of pathogen exposure to personnel and the public and/or a release to the environment. As described above, staff awareness and engagement increased through critical thinking activities and workshops. The analysis of a one-off pre- and post-test assessment using the same set of questions could contain some biases, and a further assessment of the impact of the workshop is recommended. Using risk assessment as a tool should ensure the sustainable development of biosafety capacity at the operational level.^20^ Multifactorial approaches covering epidemiology of diseases and pathogens (eg, route of transmission, susceptible hosts, host immunity, infectious doses, etc), high-risk laboratory procedures (high volume, pathogen propagation, aerosol, etc), and risk mitigation and controls should be included in trainings.^21^ Continuing education including retraining is key to raise awareness, advocate for behavioral changes, and establish a new norm of good laboratory practices.^17^ Challenges identified above were similar to the outcomes of a global gap analysis of high-containment laboratories, which revealed that in developing economic countries, operational challenges included varied numbers of safety equipment, high maintenance costs, insufficient funding to meet basic material/utilities, and imbalance of training.^22^

Self-evaluation using the LMT-S, which is recommended as a standardized self-help and training tool, provided some insight into gaps and opportunities for improving laboratory biosafety and biosecurity measures.^10^ The Wilcoxon signed-rank test was used in 11 Thai veterinary laboratories to compare the LMT-S scores between 2 years (2018 and 2016) due to the small and nonnormally distributed data set. The test confirmed that the scores of all 4 areas as well as the grand total of the LMT-S evaluation had increased significantly during the CATH2 implementation period. However, the results should only be used to monitor improvement over time of the individual laboratory center. As self-evaluation can contain biases and different assessment teams often score differently, the results should be interpreted with caution and cannot be used to compare between centers.

Biosafety breaches are not a single laboratory or a country problem when considering contagious pathogens.^3^ To achieve sustainable biosafety and biosecurity at the global level, national and international communities must work together.^23^ International guidelines and standards on biosafety and biosecurity with some forms of regulatory requirements could provide guidance at the national level.^3^ International biosafety bodies could help fill the gaps in developing countries through trainings, facility upgrades/refurbishments, and essential equipment.^22^

Considering the “One Health” concept, education on biosafety and biosecurity should be extended across disciplines working in relation to veterinary and other health professions that could potentially expose individuals to dangerous pathogens.^24-26^ Achieving sustainable biosafety and biosecurity capacity and applications of biosafety and biosecurity require flexibility, as individual facilities face unique situations and constraints. National regulatory frameworks and policies providing guidelines require nationwide harmonization and enforcement to achieve a standard competency. Laboratory personnel should be able to imply multifactorial risk-based approaches when deciding risk mitigations and selecting suitable safety equipment. For developing and low-resource countries, the focus should be on purchasing necessary safety equipment, maintaining the existing equipment, and training staff to improve biosafety competencies.
